# Lapidotherapy

**Published:** 1900-02

**Authors:** 


### LAPIDOTHERAPY.

A HANDFUL of Parisian specialists who have threshed other fields dry, have sprung a new fad on the public in lapidotherapy. As its name suggests this new treatment consists of the overthrowing of disease and pathologic conditions by the application of precious stones. It has not yet been made public how the gems are employed or what stones are used for the different diseases. Still it should be no difficult matter to apply lapidotherapy. As an illustration let us take twentieth century melancholia. The layman would designate this very common complaint as plain "blueness" resulting from lack of money and the luxuries of life. How easy it is to cure it by means of the newest therapeutics.

Such a case as the following might not be out of place in the new work reports:

Case I.—M. Pierre Tazelot, aged 30, a poor but honest porter; married; he has a wife also several children who display the same symptoms. Previous to his marriage he was of a shiftless nature, but when M. le Cure pronounced him a husband he was forced to obtain employment. From this time his symptoms began to be manifest. At the birth of each succeeding child these symptoms became greatly aggravated, and he finally came to me for treatment, after vainly trying to get relief at the old Saltpeter Hospital and the Charcoal clinic. At this latter place he was much improved during the treatment, believing himself an engaging personage of the empire. On ceasing the treatment he grandly ordered a gendarme to draw a million francs from his treasury and was immediately arrested.

When he came to me he suffered great depression of spirits. He could not satisfy his appetite. He was earning but a poor, miserable, one franc a day by working from three o'clock in the morning until midnight. When his day's work was completed he suffered great lascitude and immediately fell into a deep slumber. Owing to the time of his being forced to go to work, he could not sleep more than three hours. I at once placed him under treatment. On the little finger of his left hand I placed a large diamond of exquisite brilliancy, held in place by a band of gold, and ordered him to return to me in a week. When he returned his eyes were bright, he walked with a springy step, his clothing was good, his skin was pink with health, and he was smoking a Turkish cigarette. He informed me now that he only worked six hours a day, and could sleep eight and ten hours. His family he said had almost entirely recovered. He thanked me effusively for the great good I had done him and requested that I send my bill to him at my leisure.

In going he informed me that M. Einstein had given him 30,000 francs for the diamond. This I consider my best case. Here was a poor man with a large family, all practically starving to death, when lo! he is cured by an application of the diamond and his grave malady is no more. Truly he is a monument to lapidotherapy. I trust my American brothers who have so much money will take up the new treatment. I shall advocate the establishment of government dispensaries, of which I shall be the dispensing officer. Viola! I shall be famous and rich.

---

Dr. J. B. Coakley discussed a most timely and absorbing subject in his presidential address at the last annual meeting of the Medical Society of the County of Erie. The water supply of a great city and the disposal of its sewage are two questions that are in juxtaposition and greatly concern every inhabitant.

It was a fortunate circumstance that members of the board of public works and other city officials were present and participated in the discussion that followed the reading of the paper. They exhibited great interest in it and betrayed in their remarks a studious knowledge of all its bearings on the public welfare. No one after listening to them could question their fitness for their several places in the city government. The great question at this time is the prevention of the pollution of the water supply. The disposal of sewage is the one great problem to deal with. If the source remains uncontaminated by human waste products and other germ-infected material the supply will be healthful no matter where the intake is located. We hope that the discussion of the subject which Dr. Coakley introduced with such intelligence, judgment and fearlessness will lead to prompt action. A full report of the proceedings of the meeting is published elsewhere in this magazine. The address itself will be printed in the March edition.

---

THE pollution of the water supply of cities is attracting attention in several localities at the present time. The cities in New Jersey along the Passaic River are very much disturbed, and very properly so, over the fouling of that stream by the sewage of towns situated along its shores.

Meetings are being held and prompt action is urged at each to prevent the further development of the evil and to correct its present deplorable state. In some instances the courts have been appealed to and in others such a course is threatened

A recent outbreak of typhoid fever at Trenton, N. J., has led to an investigation and the suspicion has been aroused that its own water supply is not what it should be. Since there is no longer any question in the minds of the well-informed members of the medical profession that typhoid fever is a water borne disease, it does not take very long to locate the cause of an endemic of the malady. The people are not slow to determine in their own minds that it is not best to locate in or even visit a city in which the water supply is a dilution of sewage.

Dr. W. A. Scott, of Niagara Falls, has pointed out the cause of typhoid fever in his city, which is traced to pollution of Niagara River by the sewage of Buffalo. It will not answer for us to permit our fair name to be smirched, or for us to be threatened with damage suits, because our sewage befouls the water supply of our neighbors, to say nothing of the importance to ourselves, to our own health, of drinking pure, uncontaminated, sewage-proof water.

---

AN ASEPTIC office is something of a novelty, even in these days of ultra asepticism; yet there is one such in Buffalo. Dr. D. W. Harrington recently built an addition to his suite of offices at 1430 Main Street, which certainly as nearly approaches the aseptic state as it is possible for such an apartment to do.

Its general shape is that of an oblong square, about 14 x 20 floor area; it is built of glass, iron, copper and tile, with gable roof, heated with natural gas burning in a fireplace, and ventilated from the roof and sides. The floor is of inlaid mosaic tiling, the mantel and sides half way up are wainscoted in white glazed block tiles, the remainder of the sides and the roof are built of glass. Over the mantel rests an aquarium well stocked with fishes and supplied with running water, hanging baskets and brackets containing plants adorn the room, on a glass and iron center table sets a microscope, a lounge and easy chair in leather, together with desk, bookcase and a few other necessary articles of furniture, go to make up one of the most delightful offices for a physician that we have ever seen.

